# Vertebrate Protein CTCF and its Multiple Roles in a Large-Scale Regulation of Genome Activity

**DOI:** 10.2174/138920209788921038

**Published:** 2009-08

**Authors:** L.G Nikolaev, S.B Akopov, D.A Didych, E.D Sverdlov

**Affiliations:** Shemyakin-Ovchinnikov Institute of Bioorganic Chemistry, Russian Academy of Sciences, 16/10 Miklukho-Maklaya, 117997, Moscow, Russia

**Keywords:** CTCF, transcription factor, insulator, loop domains, gene regulation.

## Abstract

The CTCF transcription factor is an 11 zinc fingers multifunctional protein that uses different zinc finger combinations to recognize and bind different sites within DNA. CTCF is thought to participate in various gene regulatory networks including transcription activation and repression, formation of independently functioning chromatin domains and regulation of imprinting. Sequencing of human and other genomes opened up a possibility to ascertain the genomic distribution of CTCF binding sites and to identify CTCF-dependent *cis*-regulatory elements, including insulators. In the review, we summarized recent data on genomic distribution of CTCF binding sites in the human and other genomes within a framework of the loop domain hypothesis of large-scale regulation of the genome activity. We also tried to formulate possible lines of studies on a variety of CTCF functions which probably depend on its ability to specifically bind DNA, interact with other proteins and form di- and multimers. These three fundamental properties allow CTCF to serve as a transcription factor, an insulator and a constitutive dispersed genome-wide demarcation tool able to recruit various factors that emerge in response to diverse external and internal signals, and thus to exert its signal-specific function(s).

## INTRODUCTION

CTCF (also known as CCCTC-binding factor) [1] is a ubiquitously expressed vertebrate nuclear protein with numerous functions. CTCF was first detected as a protein specifically recognizing three regularly spaced repeats of the CCCTC sequence located ~200 bp upstream of the chicken *c-myc* gene transcription start site [2, 3] and binding to a chicken lysozyme silencer [4, 5]. CTCF contains three domains, one of which is a DNA-binding domain with 11 zinc fingers.

CTCF is evolutionally conserved "from Drosophila to humans" [6-8], and nearly 83-85% amino acid residues of the full-length protein are identical among human, rabbit, chicken, and frog. The identity rises up to 100 % in the zinc finger containing region [9].

The CTCF gene is expressed in multiple tissues. In the human genome it is located on chromosome 16q22.1 within the loss of heterozygosity region and is a suspected tumor suppressor gene in breast and prostate cancers [10, 11].

CTCF shows a dynamic distribution among cell compartments in a cell cycle-dependent manner. In interphase, CTCF is a nuclear protein mainly excluded from the nucleolus. During mitosis, especially in anaphase and metaphase, CTCF associates with the centrosome [12]. CTCF can be phosphorylated by protein kinase CK2 *in vivo* [13], as well as poly(ADP-ribosyl)ated (reviewed in [14]), and this latter modification is thought to regulate CTCF activity as a component of insulators [15]. In addition, CTCF is capable of activating self-modification of poly(ADP-ribose)-polymerase-1 [16]. Recently, sumoylation of CTCF was also reported [17].

Using different combinations of zinc fingers, CTCF binds diverse DNA cis-regulatory sequences [18] and participates in multiple cellular processes. A database of CTCF binding sites is constructed and now contains more than 30,000 characterized sites [19]. Binding of CTCF to a target DNA region can lead to either activation or repression of the transcription of the gene under its control [20].

Binding of CTCF to DNA can be methylation sensitive except when the binding site does not contain CpG dinucleotides. Moreover, CTCF binding can protect its sites from methylation (reviewed in [20]). This suggests the implication of CTCF in epigenetic regulation [21-23] and in X-inactivation choice and escape [23-26]. Binding of CTCF to DNA can be also regulated by nucleosome positioning [27] and *vice versa* [28].

## CTCF AS A MULTIFUNCTIONAL REGULATOR

### CTCF and Cancer-Associated Genes

The location of the human CTCF gene within the breast and prostate cancer loss of heterozygosity region on human chromosome 16q22.1 allowed Filippova *et al*. [10] to hypothesize that CTCF is a candidate tumor suppressor gene. The hypothesis was further supported by the finding of a tumor-specific rearrangement of *CTCF* exons [10] and several cancer-related mutations interfering with CTCF binding in a gene-specific manner [29]. However, a study of the CTCF mRNA level in breast carcinoma revealed no significant tumor-associated loss or decrease in expression, and therefore *CTCF* is unlikely to be a tumor suppressor gene targeted by the 16q22.1 loss in breast cancer [30]. Moreover, CTCF protein level was found to be elevated in breast cancer cell lines and tumors when compared with normal counterparts, also not suggesting that *CTCF* is a tumor suppressor gene [31]. However, the authors in [31] put forward the hypothesis that up-regulation of CTCF may be linked to the resistance of breast cancer cells to apoptosis. This suggestion was substantiated by the demonstration that overexpression of CTCF partially protects cells from apoptosis induced by the proapoptotic protein Bax. On the contrary, down-regulation of CTCF caused apoptotic cell death [31]. The experiments above thus indicate that CTCF behaves more like a regulator of other tumor-associated genes than a classical tumor suppressor. This is in accord with the observation that CTCF targets include various genes with regulatory functions, and among them the well known oncogene *MYC* [3], tumor suppressor gene *RB1* [32] and other genes (see [20]).

### CTCF and Development: Regulator of Master Regulators?

A complete knockout of the CTCF gene results in early embryonic lethality of mice indicating its essential function in early development [33, 34]. However, conditional deletion of the CTCF gene in fetal liver cells [34] and thymocytes [35] did not cause cell death but interfered with their growth and regulation. CTCF gene knockdown using antisense constructs shows inhibition of K562 cells differentiation [36] and apoptotic cell death in breast cancer cell lines [31]. Downregulation of CTCF expression by siRNA resulted in reduced expression of MHC class II genes [37]. The data above support the multifunctional nature of CTCF, and at the same time show that CTCF is not essential for cell growth in culture or for tumor cells proliferation.

CTCF regulates expression of the *Pax6* gene coding for a highly conserved transcription factor of the paired box family, which is important in central nervous system development including development of eye [38]. A knockdown of CTCF in transgenic mice enhances the transcription of *Pax6,* whereas the overexpression of CTCF suppresses *Pax6* transcription, possibly by the insulation of the *Pax6* promoter from its enhancer [39]. Also, CTCF gene transcription was moderately (2-3 fold) induced in rabbit corneal epithelial cells by epidermal growth factor in a dose-dependent manner, and in human Rb cells by serum factors, this activation also resulted in suppression of *Pax6* transcription [40, 41].

Depletion of maternal CTCF in mouse oocytes resulted in transcriptional misregulation of multiple genes, meiotic defects in the egg and mitotic defects in the embryo. The authors concluded that *CTCF* was an important maternal effect gene playing an essential role in early embryonic development [42].

The data above seem to be rather contradictory. However, they could be understood considering an essential difference between gene knockout leading to complete exclusion of CTCF from all cells of the organism and conditional gene knockout or gene transcript knockdown in a somatic tissue. In these latter cases a protein depot could exist for abundant and stable proteins, which is probably the case for CTCF. This depot might be sufficient to maintain several cell divisions, as with maternal CTCF in eggs [42]. However, there are no data allowing to estimate the CTCF content in various cells.

Collectively, these data indicate a critical role of CTCF in mammalian development. It can be argued that CTCF functions include (but are not limited to) regulation of other regulators, but the question how CTCF performs this regulation remains unsolved.

## CTCF AND GENOME FUNCTIONING

### CTCF and Chromatin Border Elements

Of all CTCF functions, the most thoroughly studied is undoubtedly formation of boundary elements in vertebrate genomes [43], which is implemented with the participation of transcriptional insulators. However, it would be incorrect to consider all CTCF-binding sequences as insulators.

There are two types of chromatin boundary elements identified so far - insulators and S/MARs. Insulators are DNA sequences that prevent activation of promoters by inappropriate enhancers and/or block the spread of condensed chromatin (for recent reviews see [44-47]). In some cases, however, these two activities can be linked to distinct parts of one and the same sequence [48, 49].

Insulators have been identified in various eukaryotic organisms, including vertebrates, *Drosophila*, sea urchin and yeast [50-54]. Some insulators can function when transferred in phylogenetically distant organisms, like sea urchin, plant and human [54, 55], and can interfere with heterologous enhancers [55, 56].

In contrast to a majority of known enhancers, the activity of different insulators can depend [52, 57, 58] or not depend [59-61] on their orientation relative to the cognate promoters. Moreover, if more than one insulator is located between enhancer and promoter, their combined enhancer-blocking activity can be much lower than that of a single insulator [62-64]. This neutralization effect can depend on the orientation of insulators relative to each other [65]. Consequently, insulators may not just subdivide a genome into domains, but rather form, in cooperation with genes, promoters, enhancers and other elements, a multilevel network regulating the transcriptional activity of the genome.

Although several reports indicated the existence of CTCF-independent insulators [66-68], CTCF participates in functioning of a great majority of these elements.

For example, CTCF plays an essential role in the activity of chicken [43], mouse [69, 70] and human [69, 71] beta-globins and chicken alpha-globin insulator elements [72], T-cell receptor alpha and delta insulators [68], an insulator of the *Igf2/H19* locus [21, 22], and other insulators [73].

Another type of DNA elements considered to be capable of forming independently regulated chromatin domains are scaffold/matrix attachment regions or S/MARs (reviewed in [74]). S/MARs are operationally defined as the DNA sequences that in *in vitro* test preferentially bind nuclear matrix or scaffold [75]. S/MARs are hypothesized to be located at the base of chromatin loops and to anchor them to the nuclear matrix thus forming structurally and functionally independent chromatin domains (for review see [74, 76]). As both S/MARs and insulators can participate in the formation of chromatin domains, their interrelation has been investigated. It was reported that the same DNA fragments, at least in some cases, display both the S/MARs and insulator properties [72, 77-81]. However, it was also demonstrated that these activities can be separated [82] or are only observed for certain genetic constructs [83].

It was found recently that the CTCF protein can be associated with nuclear matrix [84]. The authors assumed that CTCF might demarcate nuclear matrix-dependent points of transition in chromatin, thereby forming topologically independent chromatin loops. Later, it was shown [85] that both CTCF and the chicken beta-globin HS4 insulator element can be incorporated in the nuclear matrix, and, moreover, HS4 incorporation depends on the presence of an intact CTCF-binding site. Recently, nuclear lamina associated domains (LADs) of the human genome were described [86], and CTCF was proposed to participate in the demarcation of these domains. All these data suggest a possible connection between the activities of S/MARs and insulators, but the problem of this relationship has not yet been solved and requires identification and comparative analysis of a greater number of these elements.

### Genomic Distribution of CTCF Binding Sites

The publication of the human and other metazoan genome sequences opened a possibility for analysis of CTCF binding sites distribution within genomes. Using chromatin immunoprecipitation, more than 200 CTCF-binding DNA fragments were identified in the mouse genome. It was demonstrated that a considerable fraction of these fragments displayed insulator-like properties when assayed using an episome-based test [87]. Binding sites were found in intergenic DNA, in gene regulatory regions and in introns. The number of identified sites was, however, not sufficient to make a reliable conclusion about their distribution in the whole genome. An attempt to map the majority of CTCF binding sites in a 1 megabase human genome region was undertaken [88] using both *in vitro* and *in vivo* approaches. Ten binding sites were identified therein that allowed to estimate the total number of CTCF binding sites in the human genome at about 30,000. CTCF binding sites were mapped within gene introns and also within repeated elements, in particular Alu.

A whole genome chromatin immunoprecipitation followed by a detection with tiling microarrays (ChIP-on-chip) was used to identify CTCF binding sites in the human genome [89]. 13,804 CTCF binding sites were identified in the genome of IMR90 human fibroblasts. Their distribution was closely correlated with gene positions along the genome. 46% of the sites were found in intergenic sequences, 20% near transcription start sites, and 34% within genes, mostly in introns. It is noteworthy that more than 67% of CTCF binding sites occupied by proteins in IMR90 cells were also found to be occupied in human histiocytic lymphoma U937 cells, suggesting constitutive binding of CTCF to a majority of its sites [89]. Similar data was obtained by Barski *et al.* [90], who analyzed the whole genome distribution of CTCF binding sites using chromatin immunoprecipitation combined with the massively parallel sequencing (ChIP-seq) approach. More than 20,000 CTCF binding sites were identified within the genome of human CD4^+^ T-cells. About 40% of the sites were mapped within intergenic regions, 30% within genes and 30% near transcription start sites. A more detailed analysis performed later for three cell types (CD4^+^ T-cells, Jurkat and HeLa) [91] revealed that about 49-56% of sites bound to CTCF were located intergenically, 33% - intronically, and 3-4% - exonically. Most tissue specific characteristics were revealed for sites occupied by CTCF that were located near promoter regions. The occupancy was maximal in CD4^+^ T-cells (15%) and minimal in HeLa cells (7%). The authors also demonstrated that CTCF preferentially occupied boundaries of repressive chromatin regions enriched in histone H3K27me3 modification and that this occupancy was also cell type specific [91]. However, minor part of all CTCF-occupied sites were located at potential domain borders, in accord with the multifunctional nature of CTCF.

Chen *et al.* [92] used the ChIP-seq approach to identify and map CTCF and several other transcription factors binding sites across the whole mouse genome. The number of CTCF binding sites in the genome was estimated as 40,000, which somewhat exceeds the previous estimations (see above), and significantly (2-50 fold) exceeds the number of other transcription factors binding sites tested.

It was also demonstrated [90] that CTCF preferentially binds to regions containing specifically methylated histone H3 - CTCF binding regions were enriched in H3K4 (all methylation states) and H3K9me1, but not in H3K9me2 and H3K9me3. Also, an enrichment of CTCF binding sites with the H2A.Z histone variant was detected, in accord with earlier data showing preferable location of H2A.Z at chromatin boundaries in yeast [93].

A great majority of ubiquitous in many cell types DNase I hypersensitive sites are bound by CTCF [94] indicating that CTCF highly contributes to chromatin architecture and regulation.

### CTCF Mediates Long-Distance Interactions of Regulatory Sites

Application of different variants of the chromosome conformation capture (3C) technique [95] allowed to identify CTCF-dependent chromatin loops (for recent review see [96, 97]). Within the mouse *Igf2/H19* imprinted region, the loops are formed between the imprinting control region (ICR) and differentially methylated regions (DMR) [98]. This loop formation depends on CTCF binding with the maternal ICR, but its exact mechanism is unknown [99, 100]. Moreover, CTCF is necessary for the interaction of one ICR allele of the *Igf2/H19* locus on chromosome 7 with an allele of the *Wsb1/Nf1* locus on chromosome 11 [101].

CTCF may participate in the loop formation in several ways: (i) by forming dimers or oligomers able to interact with two or more different DNA regions; (ii) by interacting with other proteins capable of DNA or protein binding, and (iii) solely by CTCF-DNA interactions if a single CTCF molecule can bind at least two distant DNA regions. There is no experimental evidence for the latter case, and participation of CTCF in protein-protein interactions will be discussed below.

### Interactions of CTCF with Other Proteins

Available *in vitro* and two-hybrid assay data indicate the formation of CTCF dimers or even multimers [102, 103], but the existence of CTCF di- and oligomers *in vivo* is still to be proved.

Affinity fractionation of nuclear extract on a column with immobilized CTCF produced a Y-box binding factor 1 (YB-1), although the role of this interaction is not quite clear [104].

Another example of CTCF-binding protein is Kaiso - a zinc-finger transcription factor of the POZ (pox virus and zinc-finger) family. Interactions between Kaiso and CTCF were documented using the two-hybrid system and co-immunoprecipitation [105]. Later, it was shown that the Kaiso factor recognizes unoccupied CTCF target sequences when CTCF binding is lost due to DNA methylation [32]. Recently, it was demonstrated that CTCF can associate with the transcription factor YY1 and transactivate *Tsix* (antisense of the *Xist* gene) thus playing a key role in X-inactivation [25]. This is the first example of a proved functionally significant interaction of CTCF with another protein. It was hypothesized that the CTCF-YY1 interaction may participate in regulation of other imprinting control regions [106].

According to *in vitro* and two-hybrid system data, CTCF also interacts with Suz12, a protein component of Polycomb repressive complex 2 (PRC2), which is responsible for methylation of histone H3 lysine 27 resulting in chromatin suppression. This mechanism is possibly responsible for the suppression of the maternal *Igf2* promoters [107].

CTCF can interact with and activate automodification of poly(ADP-ribose)-polymerase-1 which, in turn, can affect activity of DNA (cytosine-5-)-methyltransferase 1 (DNMT1) and, consequently, chromatin structure [16, 108].

It was shown that the HeLa RNA polymerase II largest subunit (Pol II LS) co-immunoprecipitates with CTCF, and that CTCF and Pol II LS epitopes colocalize to the beta-globin insulator *in vivo* [109].

The *in vitro* immunoprecipitation and two-hybrid assay indicate that the SNF2-like chromodomain helicase protein CHD8 interacts through its carboxyl-terminal region with the zinc finger domain of CTCF. Using chromatin immunoprecipitation, CHD8 was also found at some CTCF binding sites *in vivo*. The authors suggested possible participation of CHD8 in CTCF-dependent insulation [110].

Using chromatin immunoprecipitation, CTCF was shown to colocalize [85] *in vivo* at insulator sites with nucleophosmin, a multifunctional acidic nucleolar protein that participates in regulation of cell growth and proliferation [111]. As nucleophosmin is a well known component of nuclear matrix [112], it was proposed that the interaction between this protein and CTCF leads to the appearance of CTCF in the cellular matrix fraction [85].

It is of interest to note that most of known CTCF-interacting partners are either transcriptional regulators (YB-1, Kaiso, YY1) or related to regulation of other cellular functions. However, it is worth noting that in most cases the data on CTCF-protein interactions were obtained by indirect or *in vitro* methods, like chromatin pull-down and two-hybrid analysis. Direct *in vivo* analysis of CTCF interactions is limited by the lack of adequate methods.

### CTCF and Cohesin Complex

Cohesin is a highly conserved multi-protein complex whose main function is to hold together sister chromatids during S and G_2_ phases of the cell cycle to ensure proper chromosome segregation. The role of cohesin in gene regulation is also emerging (for reviews see [113, 114].

Recently, when studying the genome distribution of cohesin specific binding sites in the mouse and human chromatin, it was found that in 60-70% cases cohesin and CTCF are colocalized, and CTCF depletion disrupts positioning of cohesin. Importantly, the reverse is not true, and cohesin depletion does not significantly affect the chromatin distribution of CTCF. Depletion of cohesin somewhat inhibited activity of a chicken beta-globin insulator in transient transfection assays. The authors concluded that CTCF largely determined the localization of cohesins, and cohesin is involved in insulator function [115-117]. A putative interacting partner of CTCF is the Scc3/SA subunit of cohesin [118].

### CTCF and Repeated Elements

CTCF binding sites were found to be located within repeating elements, such as the (GT)_22_(GA)_15_ microsatellite A9 in intron 2 of the HLA-DRB1^*^0401 gene [119], trinucleotide repeats, where CTCF binding modulated their instability [120], and Alu-elements [88, 121]. It was also shown that B2 repeated sequences were significantly overrepresented in CTCF binding regions of the mouse genome [122].

Interestingly, some Alu elements can function as insulators [121, 123]. Alu elements are capable of transposition, and their relocation within the genome together with CTCF binding sites may cause reorganization of the domain structure, as we proposed earlier for S/MARs-containing retroelements [74].

## REGULATION OF THE CTCF GENE

Despite the obviously important regulatory function of CTCF in multiple cellular processes, very little is known about regulation of the CTCF gene itself. Transcription of the CTCF gene was moderately (2-3 fold) induced in rabbit corneal epithelial cells by epidermal growth factor in a dose-dependent manner, and in human Rb cells by serum, the activation resulted in *Pax6* transcription suppression [40, 41]. Expression of CTCF in mice was reduced with age [124].

Treatment of human choriocarcinoma JAr cells with lithium resulted in a 2-3 fold increase in the CTCF mRNA content [125].

The CTCF promoter contains a CpG island, no TATA-box, a highly conserved YY1 transcription factor binding site and potential binding sites for GATA-1 and p53 [126, 127]. Within the chicken CTCF promoter several elements characteristic of cell cycle-regulated genes were found [126].

## POSSIBLE MECHANISMS FOR CTCF VERSATILE REGULATORY ACTIVITY

The regulatory effect of CTCF on gene transcription may involve several possible mechanisms some of which are illustrated in Fig. (**1**).

### 1) Conditional Transcriptional Insulation

**Fig. (1).**
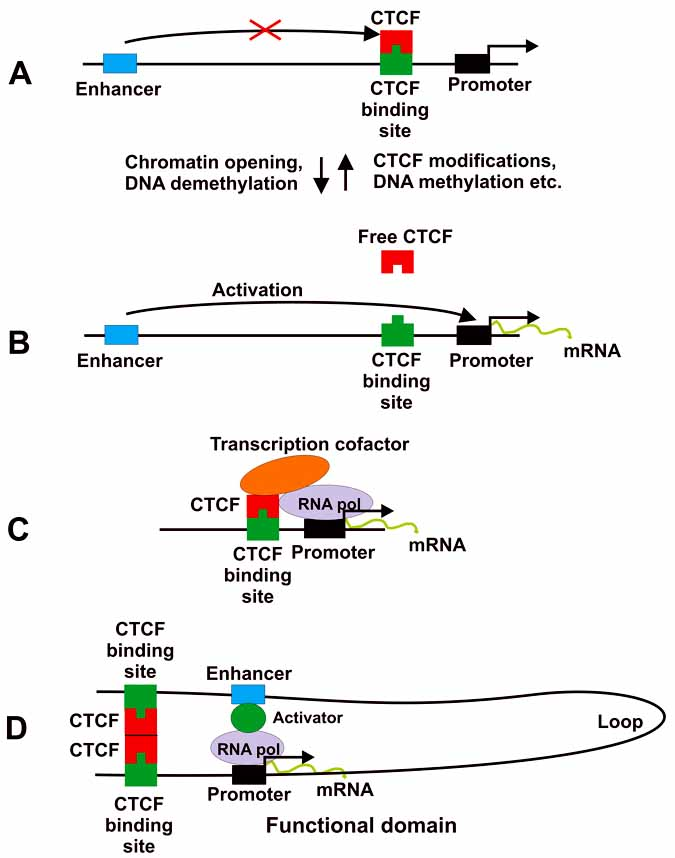
Possible mechanism of gene regulation with the participation of CTCF. See text for detail.

In this mode CTCF regulates the access of an enhancer to the promoter region *via* either CTCF binding to a vacant binding site located between enhancer and promoter (Fig. **1A**) or by release of its constitutively occupied binding site (Fig. **1B**) due to modification (e.g. phosphorylation) of CTCF or modification (methylation) of its binding site. The former type is apparently used in the regulation of the *H19*-*Igf-2* locus transcription, as well as in the *Pax6* promoter regulation [40]. The latter regulation type is based on the ability of CTCF to be subject to phosphorylation [13], poly(ADP-ribosyl)ation [14, 15] and sumoylation [17].

### 2) Genome-wide Dispersed Anchor/Demarcation Function

CTCF may serve as an anchor protein whose binding to its binding sites can recruit other trans-acting regulatory elements to allow DNA bound functional (e.g. transcription) protein complex assembly (Fig. **1C**). The abundance of CTCF and its numerous binding sites allow its participation in a multitude of functions involving the CTCF DNA binding domains and the capacity of interacting with other proteins. CTCF works as a constitutive demarcation tool of the genome/chromatin. Depending on particular genomic context and availability of various factors emerging in response to external or internal cellular signals, a particular CTCF could enable recruiting a particular set of proteins for executing a particular function. Therefore, the colocalization of CTCF with RNA-polymerase II as well as its capacity of binding various transcription factors might play a role in transcriptional regulation. This model is in agreement with constitutive binding of CTCF to a definite subset of its binding sites. Here, the induction of a transcription factor capable of interacting with promoter DNA-bound CTCF might activate the preinitiation complex.

### 3) Functional Domain Formation and Insulator Function

Finally, the capacity of CTCF to form di- or multimers and interact with other DNA binding proteins suggests its possible participation in DNA bending and directing enhancers to their cognate promoters as shown in Fig. (**1D**). In addition, two CTCF molecules in cooperation with other proteins can insulate the enhancer-promoter system from external regulatory interference.

Although all these possibilities seem quite reasonable, they certainly need additional experimental support.

## CONCLUSIONS

CTCF is a striking example of a multifunctional regulator. It participates in activation/repression of gene activity, chromatin insulation, formation of chromatin loops, X-inactivation and escape, positioning of the cohesin complex etc. CTCF regulates a number of regulatory genes including oncogenes and tumor suppressors, as well as genes regulating development and differentiation. CTCF is subject to several important post-translational modifications. CTCF is not important for single cell proliferation, but it is very important for development of multicellular organisms. The number of CTCF genomic binding sites exceeds that for most other transcription factors.

At the same time, the mechanisms underlying the CTCF functions remain largely unknown. In particular, functioning of CTCF as a component of insulator complexes and its participation in formation of loop domains implies protein-protein interactions, but the number of the CTCF-interacting protein partners found is quite low. Therefore, search for proteins and/or protein complexes interacting with CTCF *in vivo* would contribute to better understanding its role in multiple cellular processes.

In this review, we tried to formulate possible lines of studies on the variety of CTCF functions probably based on three intrinsic properties: the ability of specific DNA binding, interacting with other proteins and forming dimers and multimers. These three fundamental properties allow CTCF to serve as a transcription factor, an insulator and as a constitutive dispersed genome-wide demarcation tool able to recruit various factors that emerge in response to diverse external and internal signals, and thus to exert its signal-specific function(s).
